# Insights Informing Strategies for Optimizing the Collection of Clinical Trial Data

**DOI:** 10.1007/s43441-025-00899-4

**Published:** 2025-12-29

**Authors:** Kenneth Getz, Emily Botto, Ana Calduch Arques, Laura Galuchie, Natalia Camargo Sanmiguel, Nicole Sheetz, Zachary Smith

**Affiliations:** 1 Tufts Center for the Study of Drug Development, Tufts University School of Medicine, Boston, MA USA; 2 Novo Nordisk, Copenhagen, Capital Region of Denmark Denmark; 3 Merck & Co., Inc., Rahway, NJ USA; 4 Gilead Sciences Inc., Foster City, CA USA

**Keywords:** Protocol design, Protocol complexity, Protocol data, Clinical research data, Clinical trial design, Clinical protocol, Protocol endpoints

## Abstract

Although past research has quantified the proportion and types of procedures that support clinical trial endpoints, little is known about the volume and nature of data collected by these procedures and their impact on participant and site burden. In response, Tufts CSDD and 15 TransCelerate Biopharma sponsor companies convened to update benchmarks and gather new insights into opportunities to optimize clinical trial data collection. In all, 105 multi-therapeutic protocols with a primary completion date after 2018 were analyzed. Data volume by study and by participant were analyzed and procedures were categorized into core, standard and non-core based on the endpoints they supported. Study results show that total data volume continues to grow with 5.9 million datapoints now collected on average per phase III protocol, up 11% annually since 2020. Non-core procedures comprise 17.8% of total phase II and 16.2% of total phase III procedures per protocol, a downward trend from the proportion of non-core procedures observed in 2020. Nearly half (46%) of phase II and 35% of phase III non-core procedures gather data for exploratory and future use purposes. On average, 6.6% and 12.6% of procedures supporting core and standard endpoints from phase II and phase III protocols are deemed non-essential (i.e., procedures determined by the clinical team or protocol authors as being performed in excess of the number of times required to demonstrate a clinical outcome), and their associated data represents 8.2% to 17.1% of the total datapoints collected. Combined, nearly one-third of all procedures and datapoints collected per protocol is classified as non-core or are non-essential procedures supporting core standard/required endpoints with more than half of this data associated with clinical and patient-reported questionnaires. As much as 30% of participant and site burden is associated with non-core procedures or are non-essential procedures supporting core, standard/required endpoints. The implications of these results and potential strategies to simplify protocol designs and lower site and participant burden, including reduction in the volume of data collected by non-core and non-essential procedures, are discussed.

## Introduction

Clinical trial protocols are arguably the most intensive and extensive plans prepared by clinical research professionals in pharmaceutical and biotechnology companies. As the blueprint directing the administration of study requirements, procedures and assessments; the screening, randomization and enrollment of study participants; and the collection of clinical research data in support of study objectives, endpoints and the clinical development plan; the protocol represents the culmination of an unprecedented number of inputs and intent from clinical teams, operations and data management functions, external collaborators and senior leadership. Finalization of a late-stage clinical protocol, for example, requires dozens of clinical team meetings, an average of seven internal protocol review meetings, and input from an average of four external collaborators (e.g., scientific advisors, key opinion leaders, regulatory consultants and patient advocacy groups), over the course of several months and up to half a year [1].

Protocol design is carefully considered and highly intentional. Based on the maturity of the compound, indication, treatment and available scientific evidence, the trial design balances study objectives, prioritizes participant safety, avoids unnecessary design and executional complexities, while ensuring scientifically sound methodology. Recently released regulatory guidelines and technical requirements harmonized under international standards (ICH) aim to further improve the quality of clinical trials [2, 3].

Protocols accommodate a growing number of objectives and endpoints [4]. Phase III protocols, for example, have an average of 19 total endpoints at database lock, a 30% increase over the average observed 15 years ago [1]. During this same period, the number of procedures performed per participant visit has risen 37% in step with the growth in the number of endpoints, and the number of countries and sites engaged per protocol have increased 44% and 63% respectively [5]. Protocols are also collecting a substantially higher volume of clinical research data. Studies conducted by the Tufts Center for the Study of Drug Development (Tufts CSDD) show that between 2012 and 2020, the average total number of clinical research datapoints collected in phase III protocols increased four-fold, from 929,203 to 3,560,201 [4].

Several factors have contributed to the increasing requirements, scope and data volume of clinical trial protocols. Global drug development pipelines are targeting more challenging disease conditions – including more narrowly defined and rare disease patient communities – requiring more assessments and data to demonstrate treatment effects. Historical requirements from global regulatory and oversight agencies have increased the volume of safety, quality and compliance data collected. Additionally, growing numbers of procedures are conducted, and data is collected to support the product label and reimbursement [6].

As protocol requirements, scope and data volume have increased, clinical trial speed and efficiency have worsened. This inverse relationship has been consistently shown during the past two decades: protocols with a higher relative number of endpoints, procedures, countries and sites have longer mean enrollment durations, higher incidence and mean number of protocol deviations and amendments, and lower participant enrollment and completion rates [4].

Most clinical research professionals have found – and empirical evidence shows – protocols collect a large amount of data which may have strategic value and inform clinical teams but is not relevant to support assessment of primary and key secondary endpoints demonstrating efficacy and safety [7]. A 2012 study conducted by Tufts CSDD established a methodology to categorize procedures based on the endpoints and objectives that they support: 'Core’ procedures support primary and key secondary endpoints and objectives; ‘Standard’ and ‘Required’ procedures support baseline health and regulatory compliance respectively; and 'Non-Core’ procedures support tertiary, exploratory, miscellaneous, and supplementary secondary endpoints. This 2012 study found that non-core procedures made up 25% of total procedures performed per phase III protocol and 18% of the total performed per phase II protocol [8]. A subsequent 2020 study by Tufts CSDD saw a proportional increase in non-core procedures in phase II.

Research suggests that rising data volume and the collection of tertiary and exploratory data not only contributes to clinical trial costs but also jeopardizes study quality and places considerable burden on sites and participants. Nahm, Pieper and Cunningham, for example, found that higher data volume adversely impacts data quality due to higher error rates [9]. Research by Getz et al. demonstrated that rising protocol complexity and scope had corresponding levels of participant and site burden resulting in longer clinical trial timelines, higher levels of inefficiency and poorer recruitment and retention performance [10, 11].

Procedures and their associated data were classified as ‘non-core’ by the clinical teams and protocol authors from sponsor companies in the 2012 and 2020 Tufts CSDD studies. Additionally, a 2023 case study asked reviewers at the Food and Drug Administration (FDA) to classify protocol procedures based on the endpoints they supported. FDA reviewers classified a higher percentage of procedures as extraneous or ‘non-core’ (26% vs 18%) and they deemed that half the data collected was ‘Not Relevant’ to informing the clinical meaningfulness of outcomes for patients [12].

Taken together, the collective body of evidence suggests a compelling need and opportunity to optimize the volume of protocol data collected and its ensuing relationship with site and participant burden. Although past research has quantified the proportion and types of non-core procedures, little is known about the volume of data collected, reasons why it is collected, how it is used, and itsimpact on participant and site burden.’

Recognizing this need, TransCelerate BioPharma, Inc. and Tufts CSDD conducted a collaborative research study in 2024 and early 2025 to shed light on the underlying drivers of clinical trial data volume and to inform internal discussions and strategies to optimize protocol designs. Several major goals framed this collaborative study including: collection of the most relevant and necessary data to answer the key scientific objectives and questions; data to inform primary and key secondary endpoints; use of sound research methodologies; compliance with regulatory requirements; and minimization of site burden and participant burden.

This TransCelerate BioPharma, Inc. – Tufts CSDD collaborative study is very timely. The recently finalized ICH E6 R3 guidelines encourage more fit-for-purpose protocol designs that leverage technologies and risk-based approaches, and optimize the volume and nature of data collected while improving participant convenience and access with appropriate quality management [2].

## Methods

TransCelerate Biopharma, Inc. and Tufts CSDD convened a working group of 15 sponsor companies — each a TransCelerate member — in April 2024. The primary aims of the working group were to characterize the volume, reason and usage of data from non-core procedures as well as to assess procedures that are conducted more often than necessary (i.e., non-essential) to support core, standard and required endpoints. To our knowledge, this study is the first to collect and analyze data on ‘non-essential’ procedures.

Working group members provided extensive input during development of the data collection instrument including which protocol variables to collect and how to prioritize them (whether the variables were “need to have” or “nice to have”). When variables of interest included metrics that have not been or rarely collected in the past, working group members provided input into how the variables may be collected and formatted, or if necessary, what appropriate proxies may be used.

Among the variables collected were

- background characteristics such as phase, therapeutic area, molecule type (small molecule or biologic), orphan designation (orphan or non-orphan),
- participant population (patients or healthy volunteers),
- route of administration,
- protocol design (traditional or master),
- adaptive design (yes or no), and
- whether health authority feedback was received during protocol design.

Data on several scientific and operational characteristics were also collected:

- number of countries,
- number of sites,
- number of participants screened,
- number of participants enrolled,
- number of eligibility criteria,
- number of study arms,
- total number of procedures performed (for an average participant),
- number of unique procedures included,
- number of datapoints per participant,
- number of endpoints,
- number of adverse events of special interest.

Working group members were asked to provide additional information on each procedure performed including its core, required, standard, or non-core classification, who was responsible for performing the procedure (PI or Specialist, Other Site Staff, or Patient Reported), and the minimum number of times the procedure was required to be performed by the highest (most important) endpoint it supported.

Working group members collectively agreed on the following sampling frame to guide the collection of protocols that would be analyzed in this study:

- Retrospective protocols with a database lock (or primary completion date) after 2018.
- Phase II and Phase III protocols.
- Excludes pediatric and device trials.
- Target of 10 or more protocols for each company.
- Therapeutic areas included in each company’s sample should be representative of their respective portfolios.

Within each working group member company, clinical teams, protocol authors or others coded all of the variables in their own protocols. Several important definitions were used to guide the data coding and collection activity (refer to Table 1). Procedure classifications were defined as follows:

- Core procedures—
  - Procedures supporting primary and/or secondary objectives
  - Procedures supporting primary, key secondary, and safety endpoints
- Required procedures—
  - Procedures supporting screening requirements
  - Procedures supporting the informed consent process
  - Procedures supporting drug dispensing and compliance
- Standard procedures—
  - Routine procedures including gathering data on baseline health, concomitant medications, demographics, adverse events, and adverse drug reactions
- Non-core procedures—
  - Procedures supporting tertiary and exploratory objectives and endpoints
  - Procedures supporting supplementary (all non-key) secondary endpoints
  - Safety and efficacy procedures that are not included as an endpoint or objective
  - Procedures supporting registration
  - Procedures not tied to an endpoint or objective^1^

**Table 1:**
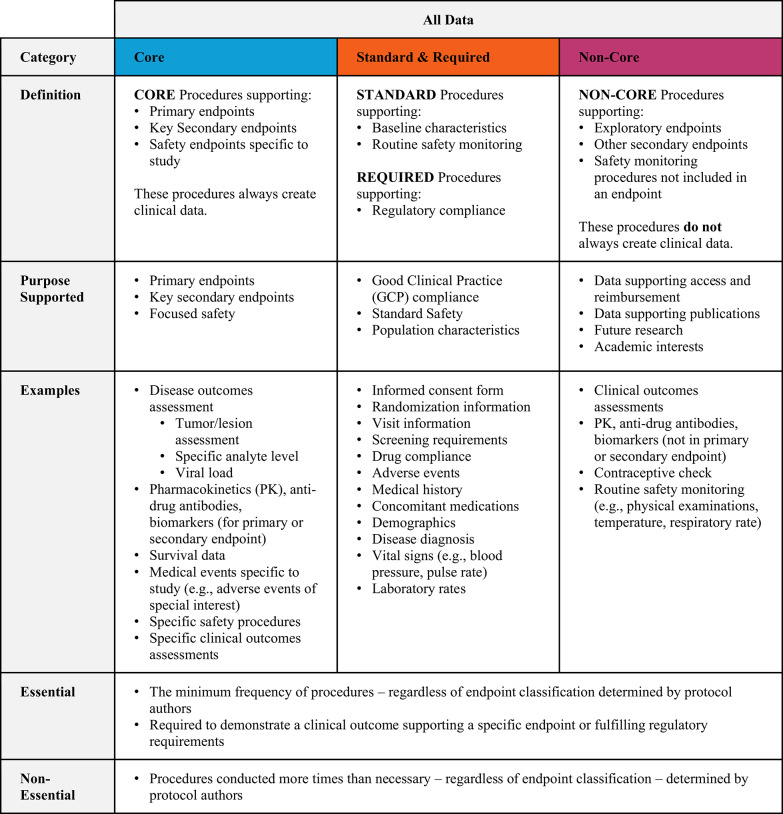
Definition of procedures generating protocol data by endpoint category

A hierarchy was used for procedures that supported more than one endpoint type. For example, if a procedure supported both a primary and tertiary endpoint, that procedure was coded based on the highest endpoint supported — in this case as ‘core’. Standard and required procedures were also considered higher in the hierarchy than non-core procedures. In all cases, when a procedure supported multiple procedure classifications, the highest type was noted.

Finally, working group member companies assessed the frequency of each procedure and determined the minimum number of times it could be conducted to address its highest associated clinical endpoint, requirement or standard. Any number of times the procedure was performed more than this amount was deemed ‘non-essential’. Table 1 describes the procedural subgroups that comprise the total data collected by a given protocol for the purposes of this analysis.

Total clinical trial data volume was calculated using two separate methodologies. For the primary method, working group members were asked to report the number of datapoints collected each time individual procedures were performed, and these datapoints were classified according to the endpoint supported by the corresponding procedure. Total data volume for trials was then calculated by multiplying the number of datapoints collected per procedure by the average number of times each procedure was conducted for each participant over the course of the clinical trial. Data volume was calculated both per participant and for the overall trial, where the number of datapoints per participant was multiplied by the number of participants in the trial. For the alternative method, working group members reported the total datapoints collected per participant over the duration of each trial. These datapoints were separated into those that were auto collected (directly into a database without work from the site, such as wearable devices); collected by the site (site staff collected and entered into database); or collected by a secondary source (collected and entered on site’s behalf by another source such as a central laboratory; or where the site had access to the data). Although it was anticipated that both methodologies would result in similar data volume estimates, this was not the case. Therefore, throughout these analyses, the primary methodology was used unless specifically stated.

For the assessment of data usage, working group members indicated whether data collected for each procedure was included in the CSR (Clinical Study Report), and whether it was analyzed and/or discussed, or merely referenced. It was also noted whether the procedure data was part of the main body or appendix of the CSR.

The data collection instrument underwent several rounds of review and revision through the spring of 2024. Working group members then piloted the data collection instrument in the summer of 2024 by providing data on 1 or 2 protocols. After the pilot, adjustments to the workbook were made, and a finalized workbook was distributed. Remaining protocol data was provided between July and November 2024. Following a comprehensive QC process to ensure high data accuracy, validity, and completeness, the final database was locked in January 2025.

Descriptive statistics (counts, percentages, means, coefficients of variation, medians, and interquartile ranges) were calculated for the dataset overall and for important subgroups. Due to differences inherent in trial design and to simplify comparisons between therapeutic area subgroups, protocols targeting cancer-related illnesses were assigned the ‘oncology’ subgroup, all others were assigned the ‘non-oncology’ subgroup.

Tufts CSDD was able to calculate participant and site procedural burden estimates using data collected in previous but unrelated Tufts CSDD studies. These studies collected perceived burden scores on a scale from 0 – 100 for approximately 60 procedures commonly conducted during clinical trials. Perceived burden scores were collected from both participants and site staff (e.g., principal investigators, study coordinators). The procedures were grouped into several larger categories – medication administration, non-invasive, invasive, lab and blood, questionnaire, routine, imaging, diary, and other. Working group members were given a list of example procedures that fall within each procedure type based on the previous research and were instructed to use these as a guideline for categorization of the procedure in each protocol. Mean burden scores were calculated for each procedure type category using the procedures included in each group. Individual procedures from the current study were mapped to the same procedure type categories and assigned the corresponding mean participant and site burden scores. Participant burden scores were further adjusted to account for procedures which could be performed at home by discounting the burden of these procedures by 10%, as has been done in previous participant burden assessments [13]. Participant and site burden score estimates were the sum of the procedural burden scores for the total number of times each procedure was performed. Total procedural burden estimates could be separated into essential and non-essential core, and essential and non-essential standard/required performances of procedures, and subgroups.

All data were collected and stored in Excel workbooks and analyzed using the statistical analysisprogram R.

## Results

Fourteen working group member companies coded and collected data on 105 protocols with primary completion dates after 2018. Table 2 provides detailed characteristics of the study data. A majority (59%) are phase III protocols, with a predominance of small molecule chemical entities represented (63%). The study data contains a mix of therapeutic areas, proportionally representative of the broad global drug development pipeline. One-quarter (26%) of the investigational treatments analyzed in the dataset target oncology indications while the remaining were non-oncology. Indications with less than 5 protocols in the dataset were grouped into “other” for blinding purposes. The study data also contains a mix of investigational therapies by route of administration with 38% oral therapies, 29% intravenous and 26% subcutaneous.

**Table 2:** Data Characteristics

	Number of protocols	Percent of total (%)
Total N	105	100
Phase II	43	41
Phase III	62	59
Small molecule chemical entities	67	63
Biologics	38	37
Oncology	27	26
Endocrinology	17	16
Immunology	14	13
Infectious diseases	12	11
Neurology	9	9
Respiratory	7	7
Other (e.g., Cardiology, GI, Hematology)	19	18
Oral	40	38
Intravenous	30	29
Subcutaneous	27	26
Other (e.g., intramuscular, ocular)	8	8

On average 17.8% of phase II procedures and 16.2% of phase III procedures were classified as non-core. Between 35.2 and 38.8% of procedures were classified as standard or required and 43.3% and 47.1% of phase II and phase III procedures as core (see Table 3).

**Table 3:**
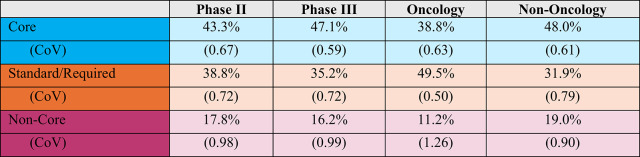
Distribution of procedures by endpoint classification per protocol, phase and TA

The incidence of non-core procedures is higher among non-oncology protocols at 19% of the total. Coefficients of variation around the mean values are also presented. Coefficients that approach and exceed 1 indicate that the individual protocol values are widely dispersed, indicating more variability between protocols of the same phase, indication and class.

**Fig. 1:**
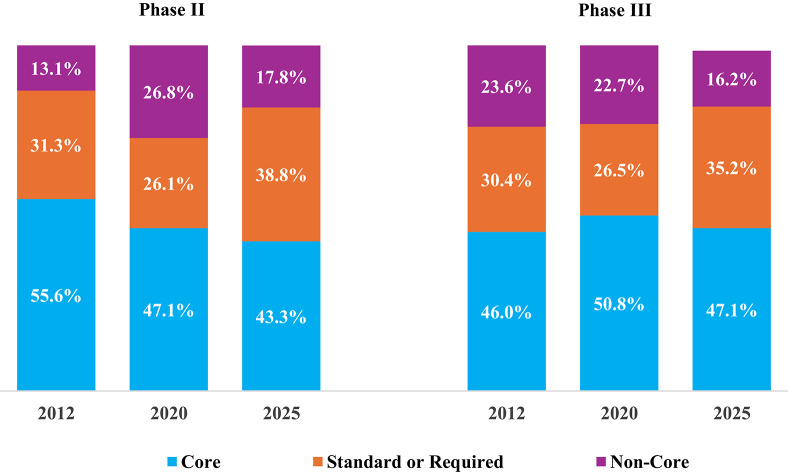
Trends in the distribution of procedures per protocol by endpoint classification

As Fig. 1 shows, the proportion of non-core procedures has declined since last measured in 2020. Phase II protocols have seen the proportion of non-core procedures drop from 26.8 to 17.8%; phase III protocols from 22.7 down to 16.2%.

Table 4 presents company-reported reasons for conducting non-core procedures. The most common reasons for both phase II and phase III studies were to gather additional safety data, future use and exploratory data. When combined, 46% of phase II and 35% of phase III non-core procedures were conducted for exploratory and future use purposes. The most common reasons for conducting non-core procedures differed when assessed by therapy area group rather than phase. For oncology studies, the most common reasons were for exploratory (43%) and future use (23%) purposes. For non-oncology studies, safety (23.5%) and future use (19.8%) were the most common reasons reported.

**Table 4:** Reasons for conducting non-core procedures

	Phase II (%)	Phase III (%)	Oncology (%)	Non-Oncology (%)
Safety	19.7	21.4	10.9	23.5
Future use	23.7	18.8	23.4	19.8
Exploratory	21.9	15.9	43.1	11.3
Quality of life/ symptom evaluation/PRO	5.3	13.3	2.9	12.5
Efficacy	5.7	10.8	10.2	8.7
Market differentiation	9.6	5.8	2.9	8.3
Support reimbursement	4.8	5.1	1.5	5.9
Regulatory request	0.4	3.1	1.5	2.4
PK/PD	2.2	1.2	1.5	1.6
Screening	0.9	0.0	0.0	0.4
Other	5.7	4.6	2.2	5.7

**Fig. 2:**
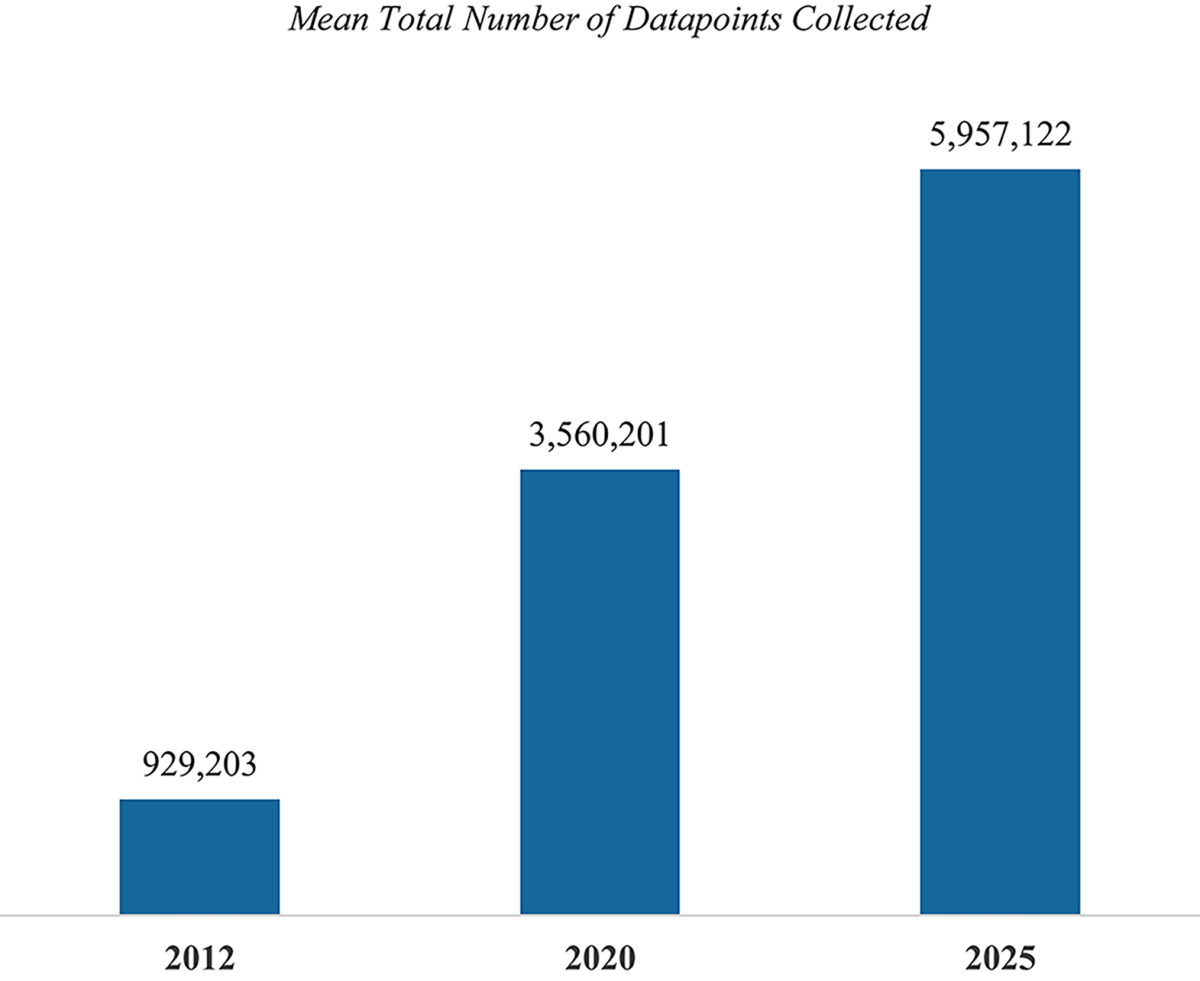
Growth in total average data volume collected per phase III protocol

Compared to benchmarks from previous studies, the total data volume per phase III protocol has risen 10.8% annually since 2020 (see Fig. 2, calculated using second data volume calculation method). Between 2012 and 2025 the volume of data collected per protocol has increased 15.4% annually.

Table 5 presents the distribution of datapoints collected per participant per protocol by procedureclassification. The majority (61.2% and 61.5%) of total phase II and phase III data collected per participant supports core procedures. Only 5.2% of datapoints gathered per participant per protocol in oncology, and 15.7% of datapoints in non-oncology support non-core procedures.

**Table 5:**
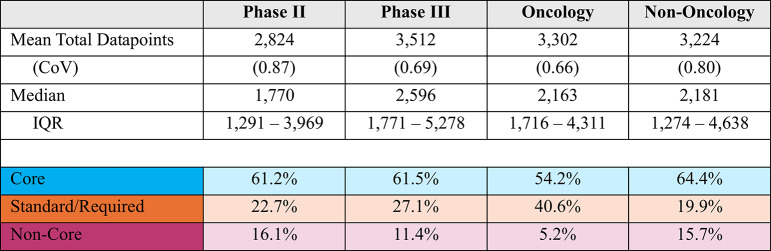
Distribution of datapoints collected per participant per protocol

When examined by phase, between 6.6 and 12.6% of core and standard/required procedures are deemed non-essential; and 8.2–17.1% of datapoints collected is from non-essential procedures (see Table 6). Combined, nearly one-third of all procedures and data collected per phase III protocol is classified as non-core or non-essential; 24.9 to 27.3% of phase II procedures and data is classified as non-core or non-essential combined. Almost four-out-of-ten (36.5%) datapoints collected per participant in non-oncology phase III protocols are classified as non-core or non-essential core, standard/required combined.

**Table 6:** Proportion of total procedures and datapoints per participant per protocol classified as non-core or non-essential

	Phase II	Phase III	Oncology	Non-Oncology
Non-essential standard/required procedures	6.6%	12.6%	11.4%	9.9%
(CoV)	(1.85)	(1.16)	(1.16)	(1.44)
Non-essential standard/required datapoints per participant	8.2%	17.1%	11.8%	14.9%
(CoV)	(1.70)	(1.10)	(1.10)	(1.29)
Non-core and non-essential core, standard/required procedures (Combined)	24.9%	31.3%	20.4%	30.6%
(CoV)	(0.87)	(0.70)	(0.85)	(0.73)
Non-core and non-essential core, standard/required datapoints per participant (Combined)	27.3%	32.5%	14.9%	36.5%
(CoV)	(1.15)	(0.58)	(0.95)	(0.65)

Data from non-core procedures and non-essential procedures is concentrated around specific procedure types (see Table 7). Questionnaires have the highest proportion of non-core and non-essential data, totaling 55.4% of data collected by questionnaires. A high relative percentage of data collected by patient diaries is also non-core or non-essential (41%), with most of this attributed to non-core procedures.

**Table 7:** Proportion of total datapoints collected from non-core or non-essential core, standard/required procedures per protocol

(Phase II and III combined) procedure type	Percent of total datapoints within type that are non-core (Column B) (%)	Percent of total datapoints within type that are non-essential core or standard/required (Column C) (%)	Total (Sum of Columns B + C) (%)
Questionnaires	37.1	18.3	55.4
Patient diaries	37.5	3.5	41.0
Invasive procedures	25.9	7.1	33.0
Lab & blood	16.0	13.4	29.4
Non-invasive procedures	10.0	17.1	27.1
Imaging	14.9	6.1	21.0
Routine procedures	4.5	11.9	16.4
Other	12.1	1.9	14.0
Medication dispensing	0.0	3.1	3.1

Invasive procedures, lab and bloodwork, imaging procedures and non-invasive procedures also collect a high relative proportion of total data from non-core and non-essential procedures.

In total, between 25 and 30% of participant burden is attributed to non-core and non-essential core, standard/required procedures with the higher relative burden found in non-oncology phase II and III protocols (see Table 8). A similar proportion of site burden is attributed to non-core and non-essential core, standard/required procedures. Non-core and non-essential core, standard/required procedures in phase II and III protocols in non-oncology contribute the highest relative burden for sites.

**Table 8:**
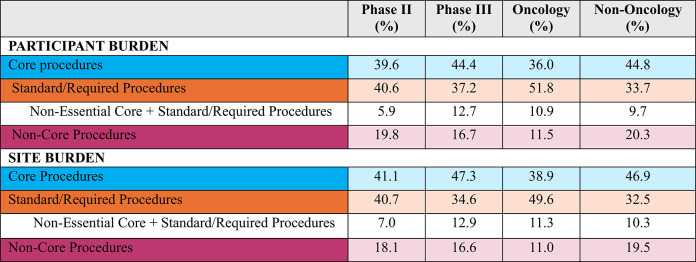
Proportional contribution to participant and site burden by endpoint classification per protocol, by phase and TA

Similar to findings in earlier Tufts CSDD studies, most data collected (74%) which support non-core procedures ultimately appear in the Clinical Study Report (CSR) (refer to Table 9). Nearly half of the data is discussed and 15.3% is referenced in the main body of the CSR. An additional 14% of data collected from non-core procedures will be either discussed, referenced or included in the CSR’s appendix.

**Table 9:**
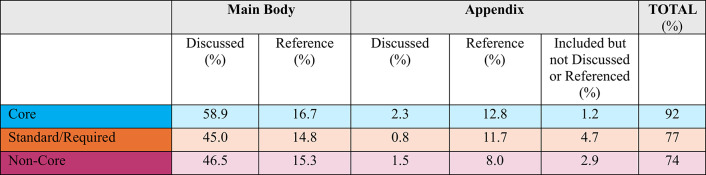
Data usage in the clinical study report (CSR)

**Fig. 3:**
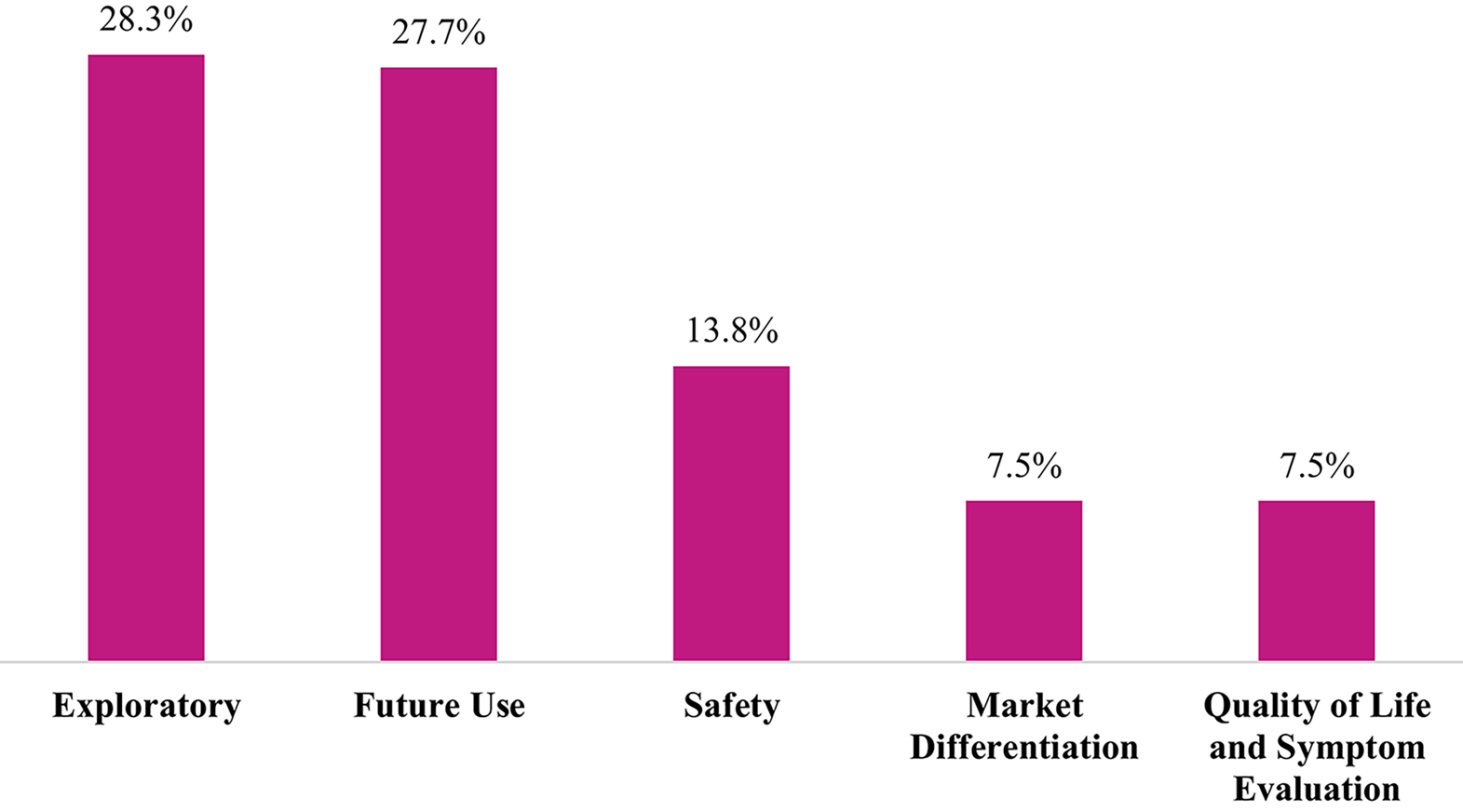
Top reasons non-core procedure data was not included in the CSR

Figure 3 shows the top reported reasons why data from non-core procedures were not used in the CSR. The majority—56%—of the data was exploratory in nature (28.3%) or intended for future use (27.7%); 14% is safety data that was collected but not core to the study.

## Discussion

The results of this study provide valuable updated benchmarks as well as new baseline measures that companies can use to compare against their own experience and identify potential areas where the volume of clinical trial data collected (and the number of procedures and assessments performed) may be reduced. The study found that protocol data volume has continued to rise since the last analysis performed five years ago. Each phase III protocol gathers, on average, 5.9 million datapoints, up 67% since 2020. This trend is due to many factors including the growing specialization of participant segmentation, rising use of biomarkers to target therapies, increasing complexity and scope of protocol designs, anticipation of questions from regulators and payers, and growing use of digital data collection technologies. Additionally, the processing power of artificial intelligence and machine learning-enabled analytical tools may be serving as a disincentive to reduce data volume [14, 15].

Phase II protocols collect a higher average proportion of total data from non-core procedures (16%) than do phase III protocols (11%). This finding may be due in part to heightened efforts to gather data in earlier phases to support the emerging safety and efficacy profile of the molecule and to inform the decision to enter later-stage clinical trial activity.

The proportion of non-core procedures has declined for both phase II and III protocols since 2020 while the proportion of standard/required procedures has increased. The number of required procedures (e.g., informed consent, adverse events, etc.) are anchored in ICH and will likely remain. However, there is an opportunity to re-assess data collection from standard or routine clinical procedures based on the disease, compound, stage of development and other factors.

This study also found that 7% of phase II and 13% of phase III protocol core, standard/required procedures are conducted more times than minimally necessary to support the highest endpoints, contributing 8% and 17% of total phase II and phase III datapoints respectively. A relatively high percentage of total participant burden and site burden is associated with these non-core and non-essential core, standard/required procedures.

Taken together, approximately one-third of all phase III and one-quarter of all phase II procedures and data collected are classified as non-core or are considered non-essential. The percentage of datapoints per participant that relate to non-core procedures or are deemed non-essential is highest in non-oncology where the combined proportion is nearly 40%. This may be explained by strategic efforts in non-oncology to differentiate the positioning of a given molecule and expand the number of target indications over time.

Procedures frequently classified as non-core and associated with a high volume of non-essential data collection represent key areas to optimize. Questionnaires, in particular, were the most common procedure that collected a disproportionately high volume of data supporting non-core and non-essential procedures. Questionnaires are often used to assess treatment impact on participants’ quality-of-life and they may be required in some but not all countries. Other procedures in this category include patient diaries, invasive procedures, lab and bloodwork, imaging and non-invasive procedures. These procedures all had disproportionately high relative volume of total data collected that supported non-core and non-essential core, standard/required procedures. The number and frequency of assessments should be balanced with their purpose and burden. Clinical teams can discuss, prioritize and weigh the impact of keeping these identified procedures (e.g., impact on enrollment timelines, site and participant burden) against not keeping them, or adjusting the frequency of the procedure.

The results of this study found that most non-core data collected (74%) ultimately appeared in the Clinical Study Report (CSR). This is not surprising as ICH E3 provides clear guidance on content. Data collected for exploratory and future use purposes was the most likely not to be included in the CSR and represents a targeted opportunity to delay or reduce data collection activity. Non-essential and non-core data that does not inform primary and key secondary endpoints and that may ultimately not be used in the Clinical Study Report represents another optimization opportunity.

This study had a number of limitations. Participating companies faced considerable difficulty coding, accessing and compiling the workbook data. As a result, some areas of analysis had relatively small sample sizes. Comparisons between results from the current and past studies should be viewed with some caution as the datasets contained different proportions of protocols by phase, therapeutic area, and protocol scope. In addition, participant and site burden are largely a function of the type and volume of protocol procedures. Future research will look at burden more expansively to include non-procedural factors (e.g., distance required to travel to the site, duration of each study visit) tobetterassess the impact of activities associated with non-core procedures. Furthermore, the classification and usage of individual data points was beyond the scope of this study since the evaluation was conducted at the procedure level. There may be additional opportunities for optimizing data collection through a more detailed assessment of the data points needed for each individual procedure. Future research will also look to conduct more granular assessments of procedures and data supporting non-core and non-essential core, required/standard procedures by individual therapeutic areas.

The findings from this TransCelerate Biopharma, Inc. and Tufts CSDD collaborative study offer valuable benchmark and baseline metrics and new insights into opportunities to optimize the dramatically rising volume of protocol data collected. While the study focused on Phase II and III trials, the implications extend across the clinical development continuum, and reinforce the importance of designing studies whose data needs are complementary and aligned throughout a development program.

The results of this study not only inform sponsor organizations in evaluating and evolving internal protocol design strategies and practices, but also inform the development of deliverables by TransCelerate Biopharma, Inc. to help sponsors consider how to balance scientific objectives, operational feasibility, site and participant burden, and regulatory requirements. Importantly, this paper does not advocate for including or removing specific procedures. Rather, it underscores the importance of mindful data collection and awareness of specific areas that may contribute to investigative site and participant burden. Clinical trials are not broken; there are clear opportunities to improve what data is collected in clinical trials, evaluated, and used. The intent is not to eliminate all non-core or non-essential procedures, but to ensure their inclusion is carefully considered and deliberate. As the complexity and volume of clinical trial data continue to grow, it is critical and timely to evaluate the value and purpose of every data point collected to reduce burden and drive more efficient, site- and patient-centric clinical research.

## Data Availability

No datasets were generated or analysed during the current study.

## Footnotes

1 Nothing in this hierarchy is intended to imply that data collection related to a tertiary or exploratory objective or endpoint, or procedures not tied to an endpoint or objective are without value or purpose to clinical research or clinical trial participants.
